# A Case of Long-Term Survival after Checkpoint Inhibitor Pneumonitis in a Patient with Squamous Cell Lung Cancer

**DOI:** 10.1155/2019/4836404

**Published:** 2019-12-26

**Authors:** Hironori Ashinuma, Satoko Mizuno, Yasushi Yoshida, Masato Shingyoji

**Affiliations:** Division of Respiratory Medicine, Chiba Cancer Center, Chiba, Japan

## Abstract

The management of grade 1 checkpoint inhibitor pneumonitis (CIP) is to withhold immune checkpoint inhibitors; however, the natural history of this condition is unknown. We herein report the case of a woman with squamous cell lung cancer who was a long-term survivor after CIP. After 4 rounds of treatment with nivolumab, a chest CT revealed a reticular pattern and ground-glass attenuation with shrinkage of the primary nodule. Nivolumab treatment was withheld without the administration of steroids. Although she remained asymptomatic, subsequent images revealed an increasing interstitial shadow until 2 months after the stop of nivolumab treatment. Thereafter, the interstitial shadow began to improve spontaneously without steroid treatment. Moreover, although the patient has not received additional therapy, disease control of lung cancer has been obtained within a follow-up period of more than 3 years. Although the exacerbation of CIP may appear on images for several months, asymptomatic cases can be followed without the administration of steroids. If the tumor had already responded prior to the onset of CIP, a favorable long-term prognosis can be expected.

## 1. Introduction

Immune checkpoint inhibitors (ICIs) have been widely used for the treatment of non-small-cell lung cancer, but attention to immune-related adverse events (irAEs) including serious events, such as pneumonitis, is necessary. The incidence of checkpoint inhibitor pneumonitis (CIP) has been reported to be between 3% and 5% in clinical settings, but a higher frequency of 19% was reported in a single retrospective study [1].

Long-term results after ICI treatment have been reported [2], but data on the natural history and prognosis of patients with CIP remain inadequate. We herein report a patient with squamous cell lung cancer who developed CIP; the patient's condition improved after the discontinuation of nivolumab treatment only, and disease control has been achieved for more than 3 years without treatment.

## 2. Case Presentation

A 64-year-old woman with a smoking history of 40 pack-years was admitted to our neurological surgery ward complaining of a headache and aphasia. A brain magnetic resonance imaging (MRI) examination revealed a 30 mm mass in the left-anterior lobe, and a chest computed tomography (CT) examination revealed a 30 mm mass in the left upper lobe with metastasis to the left hilar lymph nodes. A brain metastasis from lung cancer was suspected. A craniotomy was performed, and the presence of a squamous cell carcinoma that was negative for epidermal growth factor receptor mutation or anaplastic lymphoma kinase fusion was confirmed. Because we were unable to perform immunohistochemistry for programmed cell death 1 (PD-L1) at that time, the PD-L1 status was unknown. After the addition of localized radiation (50 Gy/25 fractions) to the excised site of the brain, she was treated with carboplatin and S-1 and achieved a partial response. After 7 months, the lung mass had increased in size. She was treated with docetaxel (DOC) monotherapy and achieved a stable disease condition. After 7 cycles of DOC, however, the lung mass began to increase in size once again.

**Figure 1:**
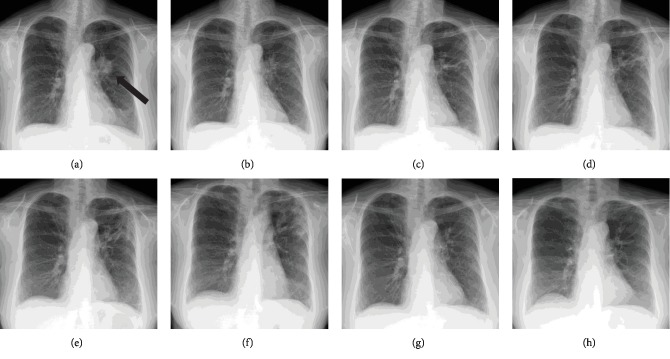
Changes in chest X-ray images. Before treatment with nivolumab (a), at the onset of CIP (b), 1 week after the onset of CIP (c), 2 weeks after the onset of CIP (d), 1 month after the onset of CIP (e), 2 months after the onset of CIP (f), 3 months after the onset of CIP (g), and 6 months after the onset of CIP (h). The relapsed lung tumor is indicated by the arrows. CIP: checkpoint inhibitor pneumonitis.

**Figure 2:**
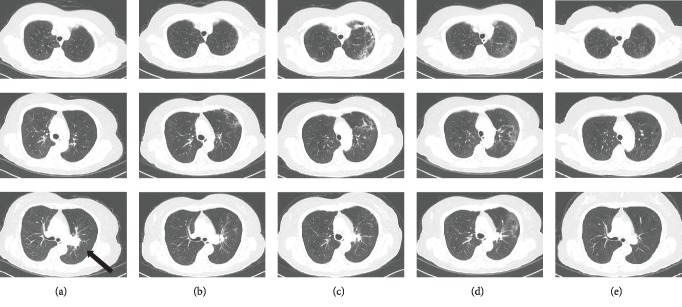
Changes in chest computed tomography images. Before treatment with nivolumab (a), at the onset of CIP (b), 1 month after the onset of CIP (c), 3 months after the onset of CIP (d), and 6 months after the onset of CIP (e). The relapsed lung tumor is indicated by the arrows. CIP: checkpoint inhibitor pneumonitis.

Treatment with nivolumab (3 mg/kg, every 2 weeks) was next initiated. On the first day of 5 cycles of nivolumab, a chest CT examination revealed a reticular pattern and ground-glass attenuation with a shrinking lung mass in the left upper lobe (Figures 1(b) and 2(b)). She had no symptoms at that time. She was afebrile, and her oxygen saturation on room air was 96%, which was the same as previous measurements. Her white blood count was 5900 cells/*μ*L, and her C-reactive protein level was 0.33 mg/dL. Other blood tests were almost normal except for a sialylated carbohydrate antigen Krebs von den Lungen-6 (KL-6) level of 606 U/mL (normal range: 500 U/mL or less) and a pulmonary surfactant protein-D (SP-D) level of 195 ng/mL (normal range: 110 ng/mL or less). Although a bronchoalveolar lavage fluid test and additional tests were not performed, she was clinically diagnosed as having grade 1 CIP. Treatment with nivolumab was discontinued, and a repeat chest X-ray and CT were performed. Although she remained asymptomatic, her chest X-ray and CT images revealed an increasing interstitial shadow similar in appearance to that of a cryptogenic organizing pneumonia pattern until 2 months after the discontinuation of nivolumab (Figures 1(c)–1(f) and 2(c)). Her KL-6 level increased to a maximum of 1300 U/mL at 2 months, and her SP-D level increased to a maximum of 302 ng/mL after one month. However, three months thereafter, the interstitial shadow began to disappear on the chest X-ray and CT images (Figures 1(g) and 2(d)), despite a lack of prednisone treatment. The KL-6 and SP-D levels also began to decrease gradually. After 6 months, almost all the interstitial shadow had disappeared and the lung mass was continuing to shrink in size (Figures 1(h) and 2(e)). Although she has not been treated with additional therapy, the lung mass has continued to shrink and no new lesions have appeared for more than 3 years.

## 3. Discussion

Here, we report a case depicting the natural history of grade 1 CIP in which disease control has been achieved for more than two and a half years without any therapy and after only 4 rounds of nivolumab administration.

In a meta-analysis, the overall incidence of CIP was 2.7% for all-grade [3]. The onset of CIP ranges from 9 days to 27.4 months [4]. Guidelines [5] for the management of grade 1 CIP recommend withholding immune checkpoint inhibitors (ICIs). If no improvement is seen, the patient should then be treated as if they have grade 2 CIP. However, the natural history of grade 1 CIP is unclear. Because of concerns over possible deterioration, steroid therapy is often administered early during the disease course. In a retrospective study where 39 out of 205 patients had CIP, all the patients with CIP received high-dose steroids [1]. Ricciuti et al. reported that patients receiving ≥10 mg of prednisone at baseline had worse outcomes than those receiving 0 to <10 mg of prednisone [6]. In a small study of melanoma patients who had ipilimumab-induced hypophysitis, patients who received a high dose of glucocorticoids had reduced survival than those who received a low dose of glucocorticoids [7]. Therefore, it is better to avoid using steroids in unnecessary cases. Predicting which cases will improve and which cases will deteriorate is difficult. In the present case, the radiological findings continued to worsen until 3 months after onset, despite a lack of clinical symptoms, and then improved spontaneously. A similar case has been previously reported [8]. In this previous report, the patient was diagnosed as having suspected interstitial pneumonia (IP) and was followed up without treatment; the IP subsequently resolved spontaneously 3 months after onset. Although details of the patient's clinical course were not provided, an improvement in grade 1 CIP generally requires several months. However, careful follow-up is still necessary. In the presently reported case, the main reason why the patient was followed without treatment was that she remained asymptomatic, despite a worsening in her radiographic findings. However, it should be noted that a fatal case of a patient who was diagnosed as having grade 1 pneumonitis but who did not initially receive steroids has also been reported [8]. Recently, Park et al. reported that the CIP patients who never needed to receive steroids had a later onset from initiation of ICIs (mean 37.48 weeks vs. 25.45 weeks), more prior lines of chemotherapy (median 2.5 vs. 1.0 lines), higher proportion of current/ex-smokers (83.3% vs. 50.0%), and fewer other accompanying irAEs (50% vs. 75%) [4]. In the present case, onset from initiation of ICIs was 8 weeks, prior lines of chemotherapy were 2 lines, ex-smoker, and no other accompanying irAEs.

Although irAEs can be lethal, the development of irAEs has been reported to be associated with a survival benefit in patients with non-small-cell lung cancer (NSCLC) [9, 10]. This survival benefit also applies to patients with pneumonitis. Fujimoto et al. reported that lung cancer patients who were treated with ICIs and developed CIP ultimately achieved higher response rates and longer progression-free survival periods than those who did not develop CIP (37% vs. 18% and 5.8 vs. 2.1 months, respectively) in a multicenter retrospective study [11]. Our presently reported case has also had a very good survival period. On the other hand, another retrospective study has shown that pneumonitis associated with cytotoxic chemotherapy or targeted therapy has an adverse impact on survival [12], which seems like a reasonable association. Therefore, the good prognosis of patients who develop pneumonitis might be specific to treatment with ICIs.

Whether the readministration of ICIs should be undertaken following recovery from an irAE remains unknown. In the presently reported case, the patient has not been retreated with nivolumab because of concerns over pneumonitis relapse and the absence of tumor regrowth. Santini et al. reported that among patients with early objective responses prior to serious irAE, the outcomes were similar regardless of whether they were retreated [13]. In cases like ours, it may be reasonable to withdraw readministration.

A limitation of this case is that we diagnosed the patient as having CIP based only on images and the clinical course. However, very little is known about the pathological findings for CIP in lung biopsy specimens, and the utility of bronchoscopy in establishing a diagnosis of CIP is unknown [14]. Although infectious diseases are an important differential diagnosis, there were no findings suggestive of an infectious disease, and the spontaneous improvement makes an infectious disease unlikely.

## 4. Conclusions

This case was able to be followed up without the steroid treatment as the patient remained asymptomatic (grade 1) though the imaging studies were initially exacerbated. This case showed response to nivolumab at the time of CIP onset, and no relapse has been observed for about 3 years without any additional anticancer treatment including readministration of nivolumab.
